# Distraction histiogenesis for treatment of Kienbock's disease: A 2- to 8-year follow-up

**DOI:** 10.4103/0019-5413.50854

**Published:** 2009

**Authors:** DS Meena, Narender Saini, Vishal Kundanani, Lokesh Chaudhary, Dinesh Meena

**Affiliations:** Department of Orthopaedics, SMS Medical College and attached group of Hospitals, Jaipur, India

**Keywords:** Distraction histiogenesis, Kienbock's disease, wrist pain

## Abstract

**Background:**

Distraction histiogenesis is known to enhance vascularity and stimulate new tissue formation. Its use in Kienbock's disease is not reported in the literature, so we proposed to study the outcome after distraction histiogenesis in treating this condition.

**Materials and Methods:**

This prospective study comprised of six patients (two male and four female) with mean age 18.16 years (range 21-35 years) with clinicoradiologically diagnosed Lichtman stage II (n = 3) and stage III (n = 3) Kienbock's disease with a mean duration of symptoms 6.67 months. The ulnar variance was neutral in two and was negative in four patients treated with the application of Joshi external stabilization system (JESS) across the wrist. The gradual distraction was done at a rate of 0.5 mm/day. After the distraction of 5-7 mm, the distractors were kept static for 3 weeks. The wrist was mobilized by using hinged distractors for next 3 weeks. Later short cockup splint was used for further 4 weeks. At the end of minimum 2 years, an assessment was done on the basis of relief of symptoms, ability to perform activities of daily living, range of movement at wrist, grip strength, and on radiology (change in the density of bone and C:MC ratio i.e ratio of carpal height to third metacarpal height).

**Results:**

The mean follow-up was of 4.5 years (range 2-8 years). The average duration of treatment was 5.3 months (range 4.5-6 months), and the duration of distraction (both static and hinged) was 8 weeks. Clinically all the patients were relieved of the symptoms with an increase in the range of wrist movement (ulnar deviation increased from 20.8° to 29.5°, radial deviation from 17.5° to 21°, dorsiflexion from 37.5° to 52.5°, and palmer flexion from 38.3° to 47.5°). At the last follow-up, activities of daily living were not affected, and all the patients were on their previous jobs without any fresh complaints. The average grip strength increased to 73-86% of normal. Radiologically the C:MC ratio (ratio of carpal height to third metacarpal height) did not show any significant improvement, but the density of lunate decreased.

**Conclusion:**

Distraction histiogenesis when used in Lichtman stage II and III with negative or neutral ulnar variance gives good symptomatic relief, allowing return to normal activities. This study has also shown that reparative process is possible in avascular bone by distraction. The authors recommend further research in this modality of treatment.

## INTRODUCTION

Various modalities to treat Kienböck's disease have been used since its description in 1910 by Robert Kienböck. These modalities range from conservative management by the immobilization of wrist to invasive methods such as radial shortening,^1^ ulnar lengthening,^2^ arthrodesis,^3^ proximal row carpectomy,^4^ and silicon arthroplasty.^5^

The controversy regarding superiority of conservative treatment over operative intervention exists in the literature. Delaere *et al.*,^6^ after their retrospective study on conservative and operative treatment, did not observe any superiority of operative management of the disease over conservative treatment. They noted that surgery was responsible for a loss of mobility of 24% and for a change in social activities in about a quarter of the patients, while grip strength was only slightly improved. Hooper^7^ also felt that the pathophysiology and the effectiveness of operative treatment remain controversial.

In the hope to find a new minimally invasive alternative treatment for this condition, we attempted to apply the principle of distraction histiogenesis as used in Perthes disease in children^8^–^11^ to revascularize the avascular lunate. It was hypothesized that distraction of the carpus and the surrounding tissue will not only unload the bone but will also increase the vascularity of the region, thus preventing any further loss of osteocytes and the increased vascularity will help in multiplication of the remaining osteocytes. This will also maintain the height of the lunate, where collapse has yet not occurred.

Hence, we proposed to study the effectiveness of distraction in Kienbock's disease on symptomatic improvement in wrist pain, range of movement and grip strength, and any radiological improvement and to assess its relation with ulnar variance.

## MATERIALS AND METHODS

This is a prospective study of six patients (two male and four female) with radiologically diagnosed Kienbock's disease in Lichtman^12^ stage II and III. The mean age was 18.16 years (range 21-35 years). Informed consent about participation in the study was obtained. The presenting symptom of all these patients was chronic wrist pain, affecting their activities of daily living. X-Rays were done to diagnose, stage the disease, and calculate the ulnar variance and C:MC ratio. Four patients had been immobilized with below elbow cast application for 1.5-2 months. Thus in two patients, distraction was used as a primary modality and in four patients as a secondary modality following immobilization. Further clinical examination of these patients was done noting the range of movement of wrist and grip strength. Radiological classification was done according to Lichtman and Degnan's classification. Ulnar variance, C:MC ratio (ratio of Carpal height to third metacarpal height), and radiolucency of the lunate was also noted.

**Figure 1:**
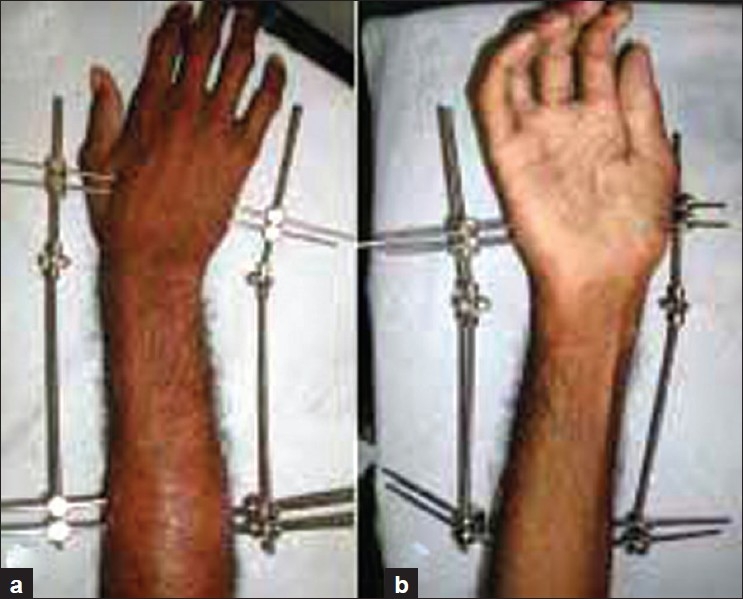
Movements of wrist with hinged distractors, thus maintaining distraction and preventing stiffness of the wrist

Based on the principal of distraction histiogenesis, uniplanar Joshi external stabilization system (JESS) distractors on both sides of the diseased wrist were applied. Pins placed under strict asepsis. Adequate exposure for pin placement was done using proper length of incision and tissue retraction to prevent soft tissue damage. Proximal two pins of 3.2 mm each were placed on the dorsoradial border of the radius and on the dorsoulnar border of the ulna. Distal pins of 2.4 mm were placed engaging the second and third metacarpals on the radial side and fourth and fifth metacarpals on the ulnar side. Wrist was immobilized in neutral position. The patient was taught pin tract care, exercises of the hand, elbow and shoulder and was also taught the distraction process. The patient was discharged with instructions for distraction at the rate of 0.5 mm/day (0.25 mm once, at 12-h interval). The patient was reviewed weekly and distraction was judged. After distraction of 5-7 mm,(the amount of distraction was judged, based on the tolerability of pain to maximum distraction), the distractors were kept static for 3 weeks. After this period, the wrist was mobilized using hinged distractors using the same pins [Figure 1]. This was continued for another 3 weeks; thereafter the distractors were removed, and short cock up splint was given in neutral position with continuation of the wrist and hand exercises for the next 4 weeks. Following this, the splint was also discarded. The rationale for the period of distraction was adopted from treatment of CTEV using JESS, which states that tissue needs to be maintained in the state of stress for at least 3 weeks for the modification to occur.^13^ Moreover in the initial patients, radiological improvement was seen by 5-6 weeks of maintained distraction, and thus a standard of 6 weeks was used in each patient for study purpose..

Assessment was done on the basis of relief of symptoms, ability to perform activities of daily living, range of movement at wrist, grip strength, and radiology. The density of bone and the C:MC ratio (normally, 0.51-0.57) was radiologically measured. Grip strength of both hands was measured by spring dynamometer. The final assessment was done at the end of 2 years follow-up from the date of surgery.

## RESULTS

**Figure 2:**
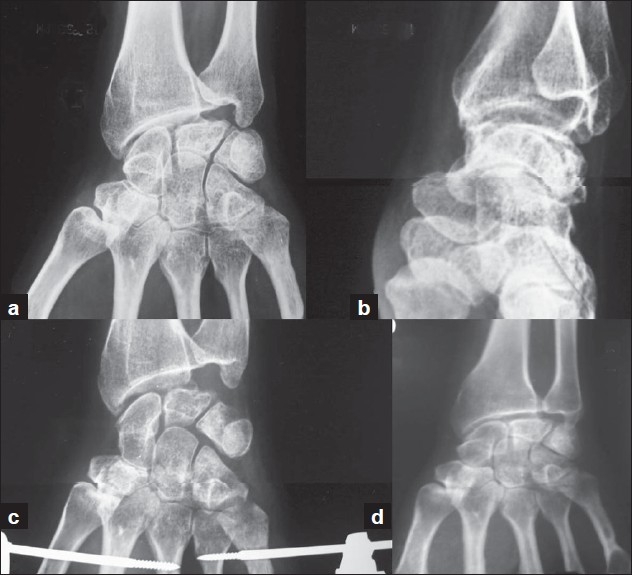
Preoperative X-rays of the wrist left side [Anteroposterior (a)and lateral (b) views] of case III with Kienbock's disease (stage II) showing normal outline of lunate with density changes. Postoperative anteroposterior views of the wrist of the same case at 8 weeks. (c) Shows maintained outline with reduced density compared to preoperative X-ray. 5 years follow-up X-rays. (d) Shows maintained carpal height with no degenerative changes

**Figure 3:**
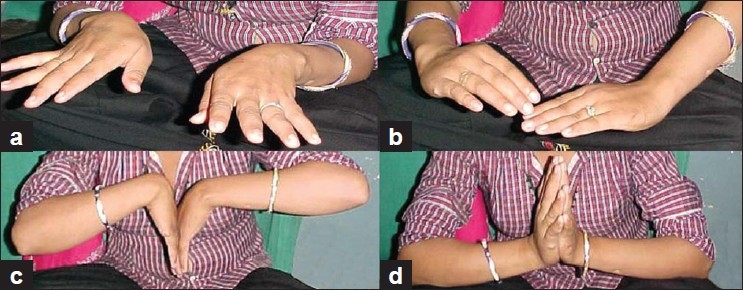
Showing preserved painless movements of wrist (left side treated) at 5 year follow-up of case III. (a) Ulnar deviation, (b) radial deviation, (c) palmer flexion, and (d) dorsiflexion of wrist

The average follow-up of our patients was of 4.5 years (range 2-8 years). The average duration of treatment from day of application of distractors to removal of last splint was 5.3 months, and the average duration of distraction was 8 weeks (the average duration of distraction includes 3 periods, one period during which the distraction of 5-7 mm was attained, then the period of 3 weeks when this distraction was maintained on static distractor and then the period of another 3 weeks when the distraction was maintained on distractors with hinges to mobilize the wrist). Clinically all the patients were relieved of the symptoms of pain and stiffness with increase in the range of movement of the wrist [Table 1]. At the last follow-up, activities of daily living were not affected and all the patients were able to return to their previous jobs without any fresh complaints. One pin tract infection (16%) was noted in our study, but was easily managed and the distraction process could be continued uninhibited. The increase in range of motion was most appreciated in ulnar deviation and dorsiflexion of the wrist. Ulnar deviation improved from an average of 20.8° to 29.5° and dorsiflexion from 37.5° to 52.5° [Table 1]. The average grip strength increased from 73% to 86% of normal. Radiologically the C:MC ratio did not show any significant improvement (pre operative 0.42 and post operative 0.43) but the density of lunate decreased [Figure 2]. Till the final follow-up, no degenerative changes were noted in the wrist despite the fact that two patients had a follow-up of 8 and 6 years, respectively [Figure 3]. The bone scan done in all six cases, postoperatively, at minimum follow-up of 2 years (range 2-8 years) revealed the reparative process in lunate and increased vascularity of the carpals.

**Table 1:** Table of patients depicting preoperative and postoperative clinical details and results

Age/Sex	Duration of symptoms (months)	Previous treatment history	Stage of disease	Ulnar variance	Total duration of treatment (months)	Duration of distraction (weeks)	Wrist movements in degree (Avg. in last column)								C:MC Ratio		Bone scan at 2 years	Complications	Follow-up (years)
											
							UD		RD		DF		PF					
														
							Preop	Postop	Preop	Postop	Preop	Postop	Preop	Postop	Preop	Postop			
24/M	6	Immobilization	III	Neutral	5.5	8	20	30	15	20	35	55	35	50	0.43	0.45	R	None	8
21/M	8	Immobilization	III	Negative	6	8	15	30	10	18	40	55	40	50	0.41	0.43	R	None	6
26/F	7	Immobilization	II	Neutral	4.5	8	25	35	20	20	40	60	45	55	0.45	0.45	R	Pin tract infection	5
35/F	6	None	II	Negative	5	8	20	22	15	15	30	40	20	25	0.42	0.43	R	None	4
22/F	5	None	III	Negative	6	8	20	25	25	28	35	50	45	50	0.41	0.42	R	None	2
26/F	8	Immobilization	II	Negative	5	8	25	35	20	25	45	55	45	55	0.4	0.42	R	None	2
							20.8	29.5	17.5	21	37.5	52.5	38.3	47.5	0.42	0.43			4.5

UD - Ulnar deviation; DF - Dorsiflexion; C:MC Ratio - Carpal metacarpal ratio; RD - Radial deviation; PF - Palmarflexion; R - Reparative process

## DISCUSSION

The ideal treatment for Kienböck's disease is either to prevent the deformity or to restore the lunate to normal appearance and function. The latter has been an elusive goal. Various treatments have been suggested for the different stages.

Conservative treatment with wrist immobilization has met with different opinions; some have reported no superiority of operative intervention over conservative management,^6^,^7^ while others^14^ have reported poor results. However, it is still accepted as a treatment in Lichtman stage I, but the dilemma is that patients are diagnosed rarely this early.

Joint leveling procedures are an accepted mode of treatment in Lichtman stage II and III. Leaving aside a few authors who have showed good results with ulnar lengthening,^15^ radial shortening has been accepted as an effective modality of treatment for these stages,^1^ but not all patients with Kienböck's disease have negative ulnar variance as reported by Ryogo Nakamura,^16^ in whose study more than fifty percent of patients had zero or positive variance. Moreover the primary contraindication to consider in the operative treatment of Kienböck's disease is ulnar-positive or ulnar-neutral variance because in patients with such variance, joint-leveling procedures (radial shortening and ulnar lengthening) cannot be performed. These procedures also change the wrist kinematics which may not be acceptable.

Revascularization procedures such as pronator quadratus muscle pedicle vascularized graft, iliac crest free graft, and dorsal flap arthroplasty procedures require pre- and postoperative angiography and demand technical and surgical expertise.^17^ Moreover, the results from long-term studies are still awaited. Silicon arthroplasty is associated with reactive synovitis and subluxation predisposing to chronic wrist pain.^18^ Radical procedures like proximal row carpectomy and wrist arthrodesis are recommended only when the disease process has advanced to a stage of intractable pain with supervening osteoarthritis.

To find out a simple, yet rational and less technically demanding procedure to treat Kienböck's disease and prevent associated complications, we at our institute studied a treatment protocol based on the principal of distraction histiogenesis using a simple external distraction device, JESS. We extended the application of the principal of distraction histiogenesis by some^8^–^11^ in the treatment of a similar condition of hip (Perthes disease). It is known that the inability of the subcortical trabeculae to sustain the increased stress, especially when devascularized, can lead to collapse. The lunate fracture may heal even with a reduced vascularity and improve the late results. Even in that group in which no initial fracture exists, the earlier the lunate is unloaded, the less collapse is to be anticipated. Thus it was proposed that by gradual distraction we will be able to unload the wrist, this will provide a biologically viable bed for the surviving osteocytes, and moreover distraction itself leads to increased vascularity thus aiding the surviving osteocytes to regenerate.

In this study on six patients followed up for a period of average 4.5 years, we found a clinical and functional improvement. There was relief in wrist pain and improvement in, the range of motion of the wrist, but there was no improvement in C:MC ratio [Table 1]. The biomechanics of the wrist was not disturbed, and no degenerative changes were noted till the final follow-up. The patients were able to return to their job and were able to carry out activities of daily living normally. It was noted that even during the treatment phase with the distractor applied, patients were able to follow their activities near normally. Some of the known complications of wrist distractor, like pin tract infection, wrist stiffness, and injury to the sensory branch of radial nerve were minimized in our series, as these were anticipated and skillfully avoided with strict asepsis, adequate exposure during pin placement with intermittent range of movement exercises, and an extensive physiotherapy protocol.

The association of a negative ulnar variance in individuals with Kienböck's disease provides the basic rationale for a radial shortening or ulnar lengthening osteotomy in the treatment of this particular condition. The results of Gelberman *et al.*^19^ showed statistically significant association between negative ulnar variance and Kienböck's disease. Later De Smet^20^ in 1994 in his findings stated that “negative ulnar variance or short ulna has been associated with Kienböck's disease, avascular necrosis of the scaphoid, and scapholunate dissociations. This correlates with our observations.

Besides one pin tract infection, there were no other complications. It was realized that adequate exposure while insertion of the pin, good asepsis, hand hygiene during pin tract dressings, and intermittent mobilization were the key factors to prevent any known complications. No secondary procedure was required.

The bone scan was done postoperatively at minimum follow-up 2 years in six patients and it showed reparative process with increased carpal vascularity. Preoperative bone scan was done in only two cases, and since there was no comparison of the pre- and postoperative scan in all cases, bone scan was not used as a criteria of assessment.

Although the number of cases is small to draw out a definitive protocol for the management of Kienböck's disease using the principal of distraction histonogenesis, however this method when used in stage II and III, with negative or neutral ulnar variance gives good symptomatic relief, with return to normal activities. This study has also shown that reparative process is possible in avascular bone by distraction, and that there occurred no degenerative changes in the wrist. The authors recommend further research in this modality of treatment.
